# Intraoperative Invasive Blood Pressure Monitoring and the Potential Pitfalls of Invasively Measured Systolic Blood Pressure

**DOI:** 10.7759/cureus.17610

**Published:** 2021-08-31

**Authors:** Sean Lam, Hong Liu, Zhongping Jian, Jos Settels, Christian Bohringer

**Affiliations:** 1 Anesthesiology, University of California, Davis Medical Center, Sacramento, USA; 2 Bioengineering, Edwards Lifesciences, Irvine, USA

**Keywords:** invasive intraarterial blood pressure, hyperresonance, damping, hemodynamics, monitoring

## Abstract

Invasive intraarterial blood pressure measurement is currently the gold standard for intraoperative hemodynamic monitoring but accurate systolic blood pressure (SBP) measurement is difficult in everyday clinical practice, mostly because of problems with hyper-resonance or damping within the measurement system, which can lead to erroneous treatment decisions if these phenomena are not recognized. A hyper-resonant blood pressure trace significantly overestimates true systolic blood pressure while underestimating the diastolic pressure. Invasively measured systolic blood pressure is also significantly more affected than mean blood pressure by the site of measurement within the arterial system. Patients in the intraoperative period should be treated based on the invasively measured mean blood pressure rather than the systolic blood pressure. In this review, we discuss the pros/cons, mechanisms of invasive blood pressure measurements, and the interpretation of the invasively measured systolic blood pressure value.

## Introduction and background

Invasive blood pressure (IBP) is the gold standard of arterial pressure measurement in 10-20% of high-risk patients [1-2]. In the remaining 80%-90% of surgical patients, the standard intermittent non-invasive blood pressure (BP) that is obtained using oscillometry with a brachial cuff has been shown to have only poor agreement with IBP in critically ill patients [3-4]. These observed measurement differences are clinically significant because they would have triggered a change in treatment in as many as 20% of the critical care patients. Non-invasive oscillometric BP measurement with a brachial cuff tends to, on average, overestimate BP during hypotension and underestimate BP during hypertension, with a significant bias and considerable scatter. Invasive BP measurement with an arterial catheter, providing continuous BP measurements, detected nearly twice as many episodes of hypotension as intermittent oscillometric measurements with a brachial cuff [5]. Continuous rather than intermittent hemodynamic monitoring is highly desirable in high-risk patients. Even when continuous BP monitoring was accomplished in medium-risk patients with non-invasive techniques, the number of episodes of intraoperative hypotension was still reduced by half when compared to intermittent monitoring with a brachial cuff [6]. Although non-invasive continuous monitoring has fewer complications than arterial cannulation, it has not yet replaced IBP monitoring as the gold standard in high-risk patients, but rather serves as an alternative in low and medium-risk patients where IBP measurements are not warranted [7].

## Review

End organ perfusion/oxygenation

An adequate blood pressure level is a means to achieve the ultimate goal of the circulation, which is adequate end-organ perfusion and tissue oxygenation. Adequate organ perfusion is mostly regulated locally, in the organs, by changing the local vascular resistance, which, when seen over multiple organs and the entire circulation, works as a re-distribution of the total flow or cardiac output (CO) [8]. In addition, the total flow or CO is also regulated centrally if this re-distribution is not enough. The local flow control via regulation of resistance of the arterioles only functions properly under the condition of adequate perfusion pressure, in which the mean systemic arterial pressure plays a central role. Continuous monitoring of local organ circulation, global flow, or CO and arterial pressure is, therefore, the key. Monitoring the microcirculation has been shown to be useful when determining the optimal BP range that is associated with adequate regulation of local blood flow and tissue oxygenation for an individual patient [9-10]. Pulse contour analysis provides a means of assessing global flow or CO because it has long been recognized that an apparently adequate BP level may not necessarily be associated with an adequate total blood flow to all the tissues [11-12]. Different organs have a different range of perfusion pressures that allow for adequate local control of organ flow. While the coronary circulation can increase flow fivefold as long as heart rate is maintained at 70 bpm, diastolic arterial pressure is maintained at adequate levels and coronary obstructive lesions are absent, the kidney is much more sensitive to decreases in perfusion pressure [13]. The average lower limit of cerebral blood flow autoregulation in normotensive adult humans is around a mean arterial pressure (MAP) of 70 mmHg [14]. Hence, the heart has a greater range of adequate perfusion pressures than both the brain and the kidneys. Blood pressure goals as adequate perfusion pressure ranges, therefore, need to be specifically determined and adjusted for every individual clinical situation by considering the patient’s specific comorbidities as well as the planned surgical procedure.

Blood pressure and surgical outcomes 

Although the real target is adequate total blood flow and adequate local flow to individual organs, most outcome data are available for blood pressure. Hypotension has been associated with increased postoperative morbidity. Even short durations of intraoperative MAP less than 55 mmHg are associated with myocardial injury and acute kidney injury (AKI) [15]. A perioperative quality initiative consensus statement also concluded that even brief durations of systolic arterial pressure <100 mmHg and mean arterial pressure <60-70 mmHg are harmful during non-cardiac surgery even without prospective studies [16]. Patients with preoperative hypertension may be more susceptible to complications from perioperative hypotension [17]. In contrast to hypotension, the degree of hypertension that is associated with harm to the patient is more difficult to define. In adult non-cardiac surgical patients, there is insufficient evidence to recommend a general upper limit of arterial pressure at which therapy should be initiated, although systolic blood pressure (SBP) above 160 mmHg has been associated with myocardial injury and infarction [18].

How is IBP measured?

IBP monitoring, in essence, replaces a small part of the wall of an artery with a stiff membrane inside a pressure transducer. To achieve this, it requires the cannulation of an artery with a stiff short catheter and the use of a short and stiff tube to connect the cannula to the transducer. In order to measure pressure, a hydrostatic reference level needs to be defined - usually, this is the level of the right atrium - and the transducer needs to be kept at the correct reference level all the time. Each component of the measurement system - transducer, hydrostatic leveling, cannula, tubing - will introduce inaccuracies or measurement errors.

Transducer

The transducer nowadays is almost always a disposable pressure transducer, which is factory-calibrated by the manufacturer. The accuracy of the disposable transducers typically is better than the accuracy required of less than ±3% or ±3 mmHg by the International Organization for Standardization/American National Standards Institution (ISO/ANSI) standard [19-20]. It needs to be zeroed, and since transducers are prone to baseline drift, this should be performed at regular intervals. In terms of quantitative error, these effects will cause a small bias of less than 3 mmHg, which is not clinically relevant in routine patient monitoring but should be considered in research or validation studies.

Leveling

The pressure transducer should be placed at heart level; by convention, this is set at the level of the right atrium. A leveling error of 10 cm will cause a measurement error of 7.4 mmHg. In clinical practice, a mean error of 3 mmHg with a standard deviation of 2 mmHg has been reported [21-22]. Again, this is probably not clinically relevant in routine patient monitoring but to be considered in research or validation studies. A more unpredictable component of leveling error is in the position changes of the operating table (rotation, tilting) where it may be difficult to maintain the proper reference position at the right atrium. It will certainly add to the overall error and is hard to quantify.

Resonance and damping

The combined system of cannula, tubing, and transducer can be seen as a second-order transmission line that guides the intra-arterial pulse wave to the transducer membrane [23-24]. This second-order system can be characterized by its natural or resonance frequency and its damping factor [25-26]. The natural frequency of the measurement system must exceed the frequency range of the arterial pulse, which extends to 20-25 Hz [23,27] or 20-22 harmonics when the goal is to accurately determine the maximum rate of pressure during isovolumetric contraction (dP/dtmax) of the systolic upstroke [28]. Higher natural frequencies can be obtained by making the cannula and the connective tubing shorter, wider, and stiffer [23,29-30]. The systems also exhibit damping, caused by friction and the viscosity of the filling fluid. Critical damping is the amount of damping required to prevent overshoot. The damping coefficient of a critically damped system is 1, however, this results in a relatively slow responding system. A damping coefficient of 0.64, sometimes called optimal damping, provides a good compromise between responsiveness and distortion. In theory, with such a damping coefficient, the amplitude is accurately measured up to 2/3 of the natural frequency, within 2%, and only shows a distortion of 6% at the natural frequency. In clinical practice, however, natural frequencies ranging from 12 to 25 Hz and damping coefficients ranging from 0.12 to 0.33 are observed [21,23,26,31-33], indicating that in clinical practice, the system is often underdamped with resonance frequencies in the same range as the frequency content of the pressure signal. An artificial increase in IBP has also been observed when the three-way stopcock is in an off-center position. On the other side, blood clots, kinking in the cannula, clamping of the arterial line tubing [34], air bubbles in the tubing, or narrow, long, or compliant tubing can cause the system to be over-damped, with damping coefficients larger than the critical damping. Whenever a dampened trace is encountered in clinical practice, the cause should be investigated. Damping will result in under-reading of SBP and dP/dtmax and over-reading of diastolic blood pressure (DBP). In under-damped situations, SBP average over-estimation was as large as 28.5 ±15.9 (mean±SD) mmHg [26] where the large scatter could be caused by the error varying with frequency and heart rate. Even adequate systems according to the criteria proposed by Gardner [35] showed an SBP over-estimation of 2.6±1.9 (mean±SD) mmHg [33].

**Figure 1:**
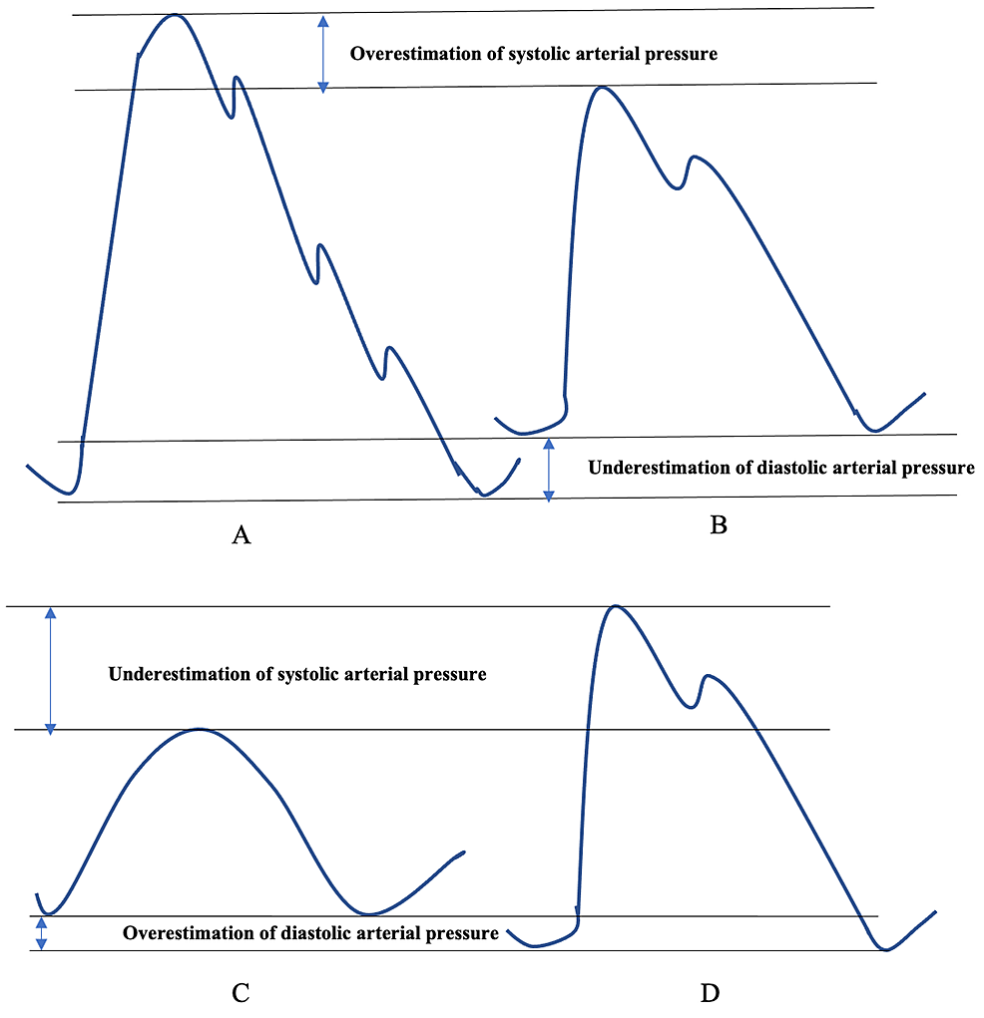
Underdamped and overdamped arterial pressure waveforms An underdamped or hyper-resonant arterial pressure waveform results in overestimation of systolic arterial pressure and underestimation of diastolic arterial pressure. An overdamped arterial pressure waveform results in an underestimation of systolic arterial pressure and an overestimation of diastolic arterial pressure. A: underdamped arterial pressure waveform with several dicrotic notches, B: optimized waveform with a single dicrotic notch, C: overdamped arterial pressure waveform without a dicrotic notch, D: optimized waveform with a single dicrotic notch "Image credit: Christian Bohringer"

Physicians need to be aware that especially the invasively measured SBP may be inaccurate in a significant number of patients and pay attention to the shape of the arterial blood pressure waveform due to damping and resonance phenomena. Wrong and potentially harmful therapeutic intervention may be undertaken by health care providers who have not been trained to recognize these resonances and damping artifacts because they will misinterpret the SBP value displayed on the monitor as the real SBP [25]. The BP waveform is a complex amalgamation of both antegrade and retrograde (reflected) pressure waves and is affected by vascular compliance, distance from the left ventricle (LV), and the 3D structure of the vascular tree [31]. The MAP is easier to measure accurately because it is less affected by damping and resonance than SBP and DBP. An under-damped, hyper-resonant trace, for example, overestimates while a damped trace underestimates SBP (Figure 1). The MAP is not significantly affected by these phenomena and is essentially the same for both traces.

Problems occur in clinical practice when a hyper-resonant IBP trace overestimates the SBP and a surgeon decides, for example, to limit the SBP to 100 mmHg when the patient is separating from cardiopulmonary bypass (CPB). If there is insufficient damping in the system, the measured SBP will be 100 mmHg while the MAP at the same time may be too low to provide adequate coronary perfusion. The patient may then have to be placed back urgently and perhaps unnecessarily onto CPB due to the erroneous overestimation of the SBP as a result of this hyperresonance artifact. The effects of resonance and damping must therefore be carefully considered whenever making treatment decisions based on the SBP. If the trace looks hyper-resonant or over-damped, the treatment decisions should be based on the MAP. If clinicians insist on making treatment decisions based on SBP then the damping within the measurement system must first be optimized before it is safe to use SBP to guide therapy. 

The industry has recognized this potential for SBP to be overestimated as a major problem and is evaluating filtering methods for acquiring radial intra-artery BP waveforms [32]. Determining the natural frequency and damping factor of the IBP measurement system for each individual patient is, however, widely regarded as too cumbersome to find acceptance in routine clinical practice. This rather labor-intensive process is mandatory in research and validation studies that seek to measure SBP accurately [32]. Algorithms that identify erroneous invasively measured BP readings have also been developed [33].

**Figure 2:**
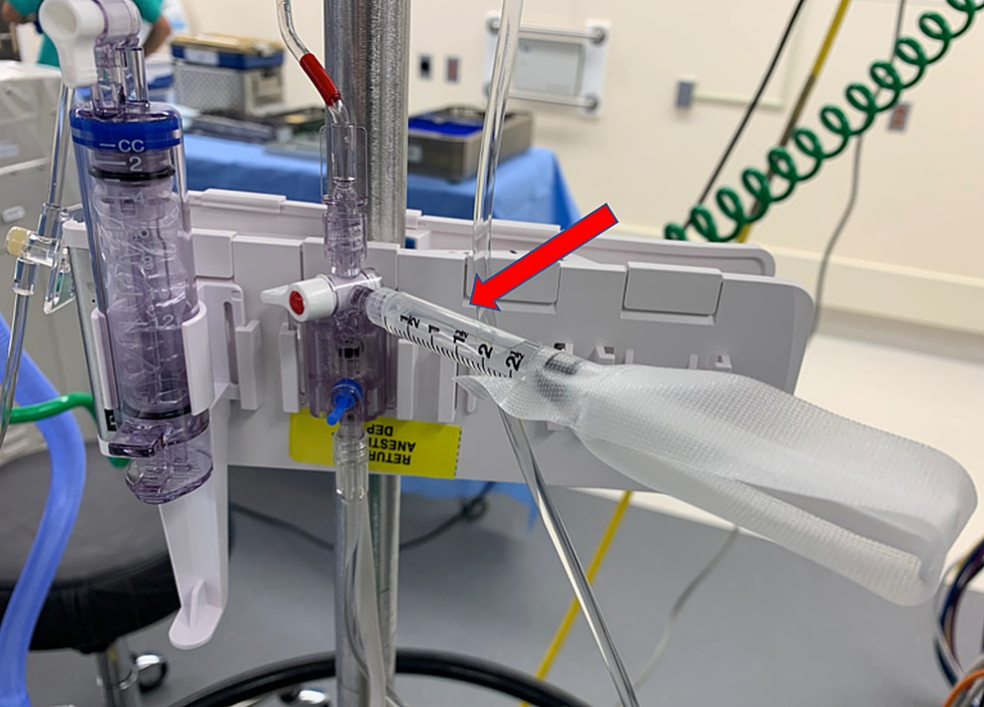
Method for eliminating hyper-resonance A syringe containing a small air bubble that is in communication with the arterial pressure measurement system allows for the dampening of a hyper-resonant arterial pressure measurement system. The larger the air bubble the greater the degree of dampening provided. "Image credit: Christian Bohringer"

Increasing the damping of a catheter-manometer system by adding a small air bubble, while increasing damping, also alters the elastic properties of the system and decreases the natural frequency, which is undesirable [35]. A method proposed by Gardner [36] to increase the damping coefficient without decreasing the natural frequency is to add a fluid-mechanical stub device containing a sealed air bubble. One of the commercial devices using this principle, the Resonance OverShoot Eliminator (ROSE) device, has been shown to increase the average damping coefficient from 0.2 to 0.8 while not reducing the natural frequency [37]. These devices, however, never were broadly adopted in clinical practice. A simple setup involving a syringe with a small air bubble in communication with the arterial line allows for the dampening of a hyper-resonant system in clinical practice (Figure 2).

**Figure 3:**
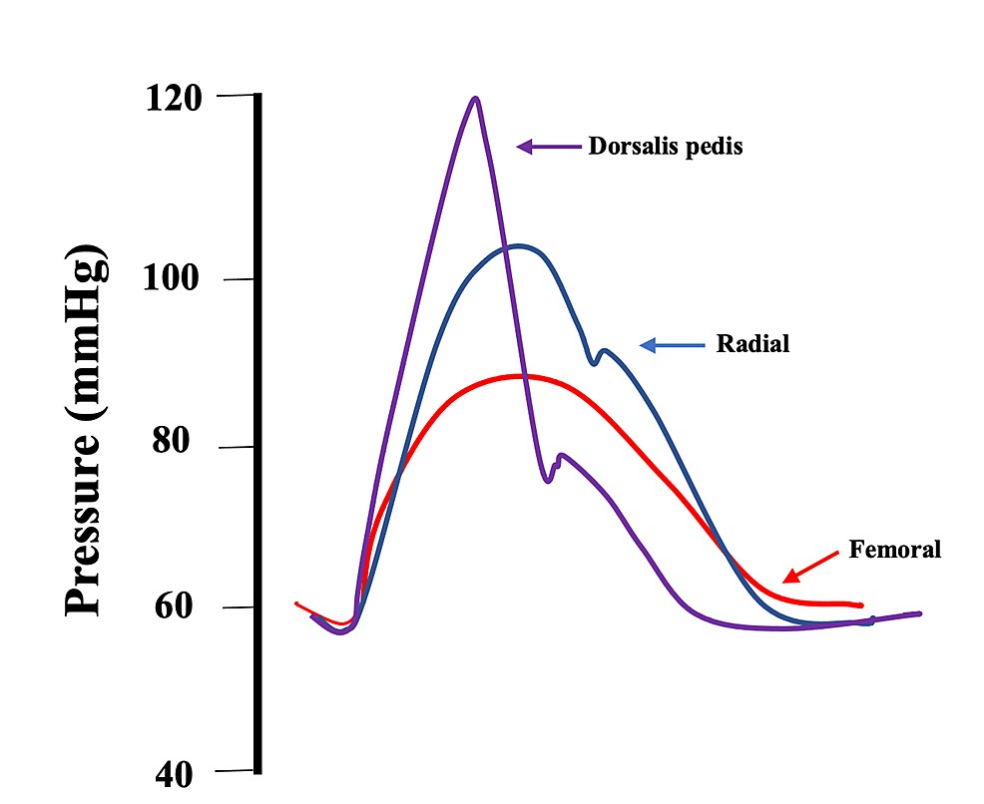
Variation of arterial pressure by site of measurement Simultaneously measured arterial waveforms from the dorsalis pedis artery, the radial artery, and the femoral artery. Systolic pressure is increasing as the monitoring site is further away from the heart while diastolic and mean arterial pressures are much less affected by the site of measurement.

Cannulation of the radial or dorsalis pedis arteries are the preferred sites of measuring IBP because the palmar and plantar arches allow for collateral blood flow to the hand and foot. This is of great importance whenever the cannulated artery develops thrombosis, usually after the arterial catheter has been in situ for a long period of time. The radial and dorsalis pedis monitoring locations protect the limb that is invasively monitored from potential ischemic damage. Patients with scleroderma should not be monitored with a radial arterial line because of a greatly increased risk of ischemic damage [38]. Brachial artery cannulation has recently gained in popularity especially in cardiac surgery and some studies have documented a low incidence of ischemic problems with this approach [39]. However, there are some reports of ischemic injuries associated with brachial arterial lines [40-41]. On the other hand, femoral artery cannulation has been associated with higher infection risk than other sites as well as pseudoaneurysm formation [42-43]. SBP tends to increase when measured at an increasing distance from the heart [44]. The site of arterial cannulation along the vascular tree is an important determinant of SBP [45-46]. A dorsalis pedis arterial line will typically show a higher SBP than a radial line, which in turn will measure a higher SBP than a femoral arterial line (Figure 3).

This phenomenon occurs because of the complex summation and reflection of pressure waves traveling over the arterial tree. The MAP is again much less affected by this phenomenon than SBP. The site of measurement of IBP must be taken into consideration when treating a patient according to SBP targets. This phenomenon is yet another reason why treating MAP is more foolproof than making treatment decisions based on SBP measurements. The measurement of MAP is not only less affected by damping and resonance but also by arterial catheter location than SBP. Using the MAP to guide hemodynamic therapy can help to avoid mistreating patients based on erroneous values that are prominently displayed on the monitor for everyone to see but that did in fact originate from measurement artifacts.

The arterial pressure signals measured by the pressure transducer are typically converted from analog to digital signals for further processing, with high enough analog-to-digital quantization resolution (such as >10 bit) and high enough sampling rate (such as 100 Hz). The digital signals might be further filtered for artifact rejection and then individual beat might be detected to calculate the displayed SBP, DBP, and MAP, with SBP being the maximum pressure in each heartbeat, DBP being the end-diastolic pressure, which usually is the minimum pressure, and MAP being the average of all pressure during the heartbeat.

Determinants and importance of SBP

The SBP is determined by the stroke volume, the duration of LV ejection, arterial compliance, the pressure wave in large arteries, and the vasomotor tone in peripheral arteries that regulates the reflection of the pressure waves [31]. When the heart contracts, it wants to eject additional volume into the proximal aorta: when the aortic valve opens, it sees a proximal aorta that has an impedance to further filling (compliance of elastance), which is already filled with blood (inertance) and which is already pressurized to end-diastolic pressure of the previous beat. While ejecting the stroke volume, the pressure in the proximal aorta rises from this end-diastolic pressure of the previous beat to the maximal pressure during the ejection phase, systolic pressure. The end-diastolic pressure is the threshold that the contracting ventricle needs to overcome in order to open up the aortic valve. The back-pressure that the heart needs to overcome during ejection or systole increases from diastolic to systolic pressure. The aortic compliance, inertance, and back pressures together form the dynamic afterload that the heart sees: the input impedance of the aorta [47].

The stroke volume (SV) is generated during about one-third of the cardiac cycle, during systole. During the entire heartbeat, however, the outflow of each SV to the periphery and to the organs, or perfusion flow, is guided by the perfusion pressure or MAP and total resistance to outflow, or systemic vascular resistance (SVR). The LV dP/dtmax has been classically considered as a marker of the inotropic state of the LV myocardium [48]. However, since it requires direct measurement of LV pressure, peripheral dP/dtmax, such as femoral or radial dP/dtmax, have been suggested as feasible surrogates for LV dP/dtmax [49]. Since the arterial pressure results from the combined interaction of the LV ejection and the arterial system properties, other potential factors such as afterload could also contribute to the peripheral dP/dtmax [50]. Nevertheless, contractility changes are the most prominent factors contributing to the arterial dP/dtmax [51].

Pulse pressure (PP) in the aorta is the result of ejecting the SV into the aortic compliance [52]. The PP, when it travels along the arterial tree, increases with increasing distance from the heart and this is thought to result from the interaction of forward waves and the reflection of pressure waves from distal sites [46]. As age increases, the walls of the aorta and the large elastic arteries progressively stiffen due to degenerative phenomena. This leads to a reduced capacity of the arterial wall to distend during systole with a consequent rise in both systolic and pulse pressure [30].

During the perioperative period, hypotension and tachycardia are associated with more adverse events than hypertension. Hypotension is a far more prominent concern in perioperative than in primary care [20]. Intraoperatively measured low radial artery SBP, MAP, and PP were associated with myocardial and renal injuries. In contrast, the correlation between diastolic hypotension and tissue injury was low [28]. This shows that MAP is equally good as SBP and PP at predicting perioperative complications. The MAP can be safely substituted for SBP for making treatment decisions when hemodynamically monitoring patients in the perioperative period whenever resonance and damping artifacts do not allow for an accurate determination of SBP.

## Conclusions

The IBP is currently still the gold standard for the measurement of arterial BP, although it comes with different sources of error. Accurate SBP is difficult to measure in routine clinical practice, mostly because of problems with hyperresonance or damping within the measurement system. The shape of the arterial pressure waveform needs to be carefully assessed for the presence of these phenomena prior to making treatment decisions based on the SBP. In addition, the SBP rises with increasing distance from the heart, and the location of the arterial line needs to be taken into consideration when interpreting SBP measurements. Making treatment decisions based on the MAP when monitoring IBP in routine clinical perioperative medicine is an approach less prone to error. Titrating treatment according to the MAP is also less labor-intensive and may avoid mistreating patients based on erroneous values that resulted from measurement artifacts.
